# A Scoping Literature Review of Digital Measures and Patient-Reported Outcomes for Refractory/Unexplained Chronic Cough: Assessment of Clinical Trials and Real-World Clinical Practice

**DOI:** 10.1007/s00408-026-00928-5

**Published:** 2026-09-10

**Authors:** Elizabeth P. Skinner, Rafael Alfonso-Cristancho, Atish De, Brian Leinwand, Elizabeth A. Duncan

**Affiliations:** 1 GSK, Durham, NC USA; 2 GSK, Upper Providence, PA USA; 3 Trinity Life Sciences, Waltham, MA USA

**Keywords:** Chronic cough, Cough endpoints, Digital measures, Review

## Abstract

**Purpose:**

Refractory/unexplained chronic cough (RCC/UCC) is a disease associated with substantial clinical and humanistic burden. A variety of endpoints have been used in RCC/UCC research to measure cough outcomes and impact on patients’ quality of life (QoL), including clinical measures of cough using digital devices, or digital measures, and patient-reported outcomes (PROs). The aim of this review was to determine which endpoints are currently used in studies of RCC/UCC, and to explore the recent evolution of new digital measures used across research and clinical settings.

**Methods:**

We conducted a scoping literature review (January 1, 2019–October 31, 2024), assessing studies of adults diagnosed with chronic cough (refractory/unexplained or newly diagnosed). Clinical trials and observational/real-world studies were included that evaluated objective and PRO measures of cough frequency, severity, symptoms, other cough characterization, and QoL.

**Results:**

Overall, 70 studies (29 manuscripts, 35 clinicaltrials.gov entries, 6 posters/abstracts) were included: 36 clinical trials (Phase 2, 3, or 4), 34 observational/real-world studies. Objective cough frequency was the primary endpoint in 24/36 clinical trials. In contrast, objective cough frequency was reported in 10/34 observational/real-world studies. Observational/real-world studies had a greater variety of endpoints for cough outcomes and PROs compared with clinical trials.

**Conclusions:**

Clinical trials and observational/real-world studies of chronic cough and RCC use a wide range of outcome measures, including objective measures of cough frequency using digital devices (digital endpoints) and subjective PROs. New approaches to digital endpoints and PROs are being developed as the study and treatment of cough evolves.

**Supplementary Information:**

The online version contains supplementary material available at https://doi.org/10.1007/s00408-026-00928-5.

## Introduction

Chronic cough is defined in adults as a cough lasting more than 8 weeks [1, 2]. People living with chronic cough may cough >400 times per day and often report sudden coughing bouts or fits, which are associated with substantial disease burden [3–5]. Chronic cough impacts overall quality of life (QoL), with substantial psychosocial and physical effects (e.g., depression, anxiety, fatigue or shortness of breath) reported in qualitative studies [6–9]. Refractory chronic cough (RCC) is a disease identified in a subset of people who experience chronic cough despite adequate treatment for known cough-related etiologies (e.g., asthma, gastroesophageal reflux disease, or upper airway cough syndrome) [1, 2, 10]. Where cause for RCC cannot be determined, despite adequate investigation with diagnostic tests and trials of therapy, it is referred to as ‘unexplained chronic cough’ (UCC). Poor recognition and under-recording of RCC in primary care can result in a burdensome diagnostic journey, with frequent treatment failures [11–14].

Suitable measures of cough must be used consistently in clinical trials and real-world studies to characterize the burden of chronic cough (including RCC/UCC) and better understand outcomes for patients. Studies may use digital endpoints, which are objective measures of cough frequency using ambulatory, wearable and smartphone digital devices. Examples of these include the VitaloJAK (Vitalograph, Buckingham, UK) [15, 16], Leicester Cough Monitor (LCM) [16–18], Strados Resp Biosensor (Strados Labs, Philadelphia, USA) [19], SIVA-P3 system (SIVA Health, Zurich, Switzerland) [19, 20], or the Hyfe Cough Monitor (Hyfe, Wilmington, DE, USA) [19, 21]). Additionally, studies may use patient-reported outcome (PRO) measures [22, 23] to measure cough severity and impact on QoL.

The landscape of endpoints for measuring cough is evolving [24, 25]. In general, clinical trials designed to demonstrate efficacy of a new treatment for RCC/UCC often prioritize objective cough frequency as a primary endpoint. However, this only has a moderate correlation with existing PROs as patients’ QoL is likely influenced by more than cough frequency [24, 26]. This reinforces the need for endpoints that capture the full scope of cough: both digital measures for objective frequency and properties of cough (e.g., timing, clustering, etc.), and patient-reported impact on QoL.

Guidance on the use of digital endpoints in research is still emerging [27–29]. Current Food and Drug Administration (FDA) guidance on new digital health technologies for remote data acquisition highlights the importance of verification (ensuring the device functions as intended), validation (ensuring the device measures appropriately for trial-specific goals) and qualification (reliability for measuring a specific clinical event) processes aligned to endpoints with clinically meaningful outcomes [29]. The FDA recommends adhering to frameworks such as the Qualification Process for Drug Development Tools (DDTs) and ensuring consistency with current Good Clinical Practices guidelines [30].

We conducted a scoping literature review to understand the endpoints used in clinical trials and observational/real-world studies of RCC/UCC, and explore the recent landscape and evolution of digital endpoints in research and clinical settings.

## Methods

### Scoping Literature Review

Searches of PubMed, Clinicaltrials.gov, and congress websites were conducted for articles published between January 1, 2019 and October 31, 2024. This window was chosen to encompass the evolving nature of new technologies to monitor chronic cough; it was determined that a longer window would disproportionately include studies lacking these technologies and potentially skew the findings. A protocol for this scoping review was not registered.

#### Eligibility Criteria

Eligible studies included clinical trials or observational studies that enrolled adults (≥18 years) with a diagnosis of chronic cough or RCC/UCC; studies which did not specify a diagnosis of chronic cough in their patient inclusion criteria were excluded. Interventions/comparators were not restricted. All eligible clinical trials and retrospective/prospective observational studies conducted between January 1, 2019 and October 31, 2024 were included.

#### Information Sources and Search Parameters

The literature search was conducted on October 31, 2024. Sources used for the literature search to best identify recently published studies of chronic cough, as well as ongoing trials, included PubMed, Clinicaltrials.gov, and congress websites. Each source was searched for “chronic cough” and other relevant key words. For PubMed the terms chronic cough [Title/Abstract] AND (clinical trial) or (interventional) or (observational) or (real-world) or (real world) or (prospective) or (retrospective) or (cohort) or (cross-sectional) or (registry) or (longitudinal) from 2019–present (October 31, 2024) were searched.

Ancillary title and abstract searches with “chronic cough” were searched for the terms embarrassment, anxiety, depression, cough bouts, continuous monitoring, incontinence, insomnia, sleep. Product terms searched included Hyfe, Strados, C-mo, SIVA and VitaloJAK. Cough episode terms used were “bout”, “epoch”, “spasm”, “fit”, “explosive episode”, “explosion”. The Clinicaltrials.gov indication searched were “Chronic Cough” and Other Terms (with no search for indication) including Hyfe, SIVA, C-mo, Strados and VitaloJAK.

Gray literature was identified through a search of key congresses (American Thoracic Society, American Cough Conference and European Respiratory Society) using key words “cough” and “chronic cough” over 2023 and 2024. Search terms for “refractory chronic cough”/ “RCC”, and “unexplained chronic cough”/ “UCC” were used to prioritize studies for review.

#### Data Charting Process and Critical Appraisal of Individual Sources of Evidence

**Fig. 1:**
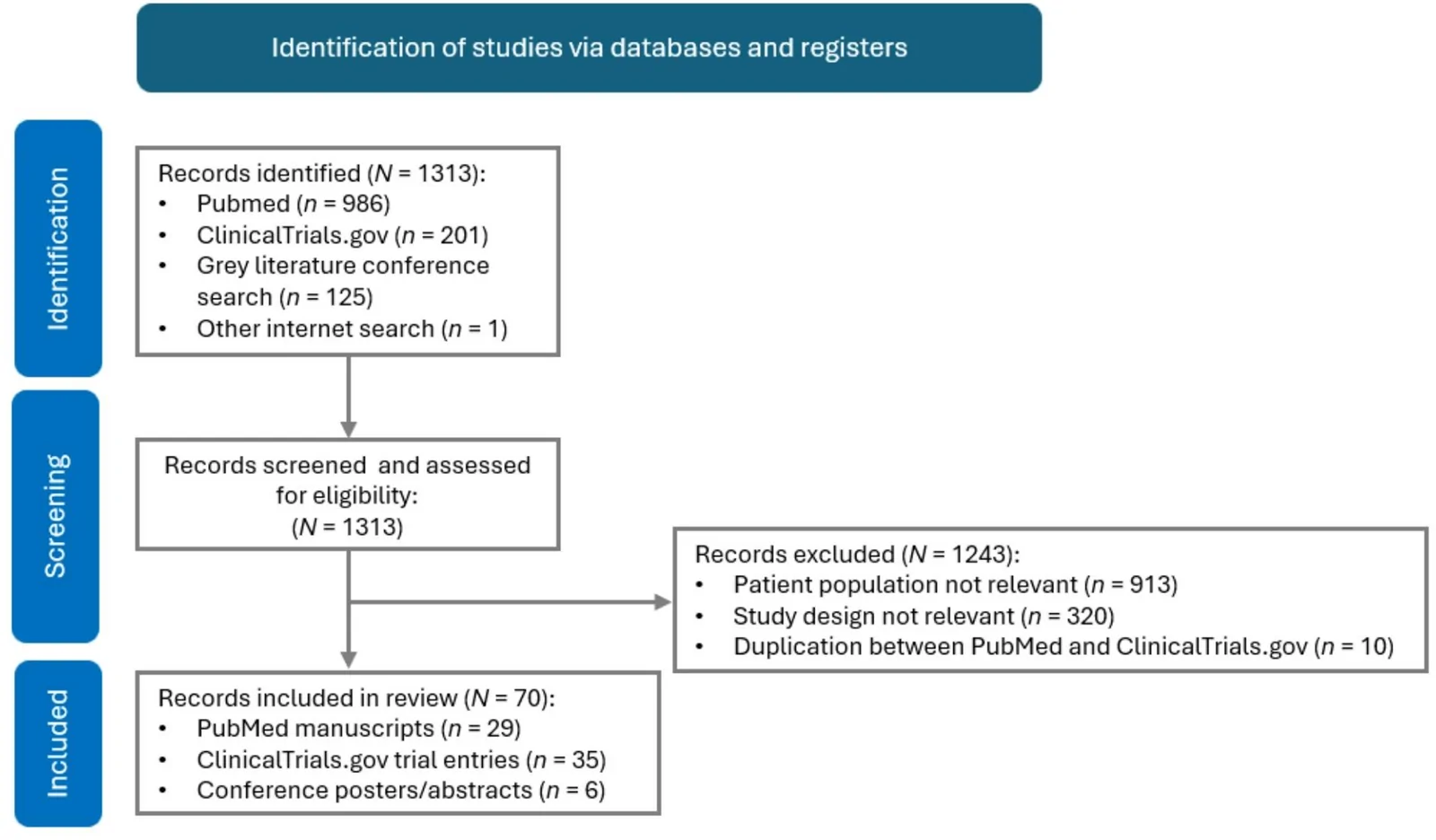
PRISMA diagram showing screening process and study selection. PRISMA, Preferred Reporting Items for Systematic Reviews and Meta-Analyses

Search results were first screened at the title/abstract level for relevant endpoints. Full texts of relevant, de-duplicated sources were then evaluated for key parameters (Fig. 1). Data extracted from the included studies were synthesized descriptively. Studies were grouped according to the outcomes assessed, and the overall number of studies was summarized and categorized by publication year. The guidance in the Preferred Reporting Items for Systematic Reviews and Meta-Analyses (PRISMA) extension for scoping reviews was followed [31].

## Results

### Scoping Literature Review

#### Search Results

The search identified 1313 publications. During screening and assessment of eligibility, 1243 publications were removed due to duplication or ineligibility (Fig. 1). Overall, 70 unique studies (comprising 29 manuscripts, 35 Clinicaltrials.gov entries, and 6 posters/abstracts) were selected for inclusion in the scoping literature review (summarized in Supplementary Table 1). Of these studies, 36 were clinical trials (Phase 2, 3, or 4) and 34 were observational/real-world studies.

#### Endpoints in Clinical Trials and Observational/Real-World Studies of RCC/UCC

**Fig. 2:**
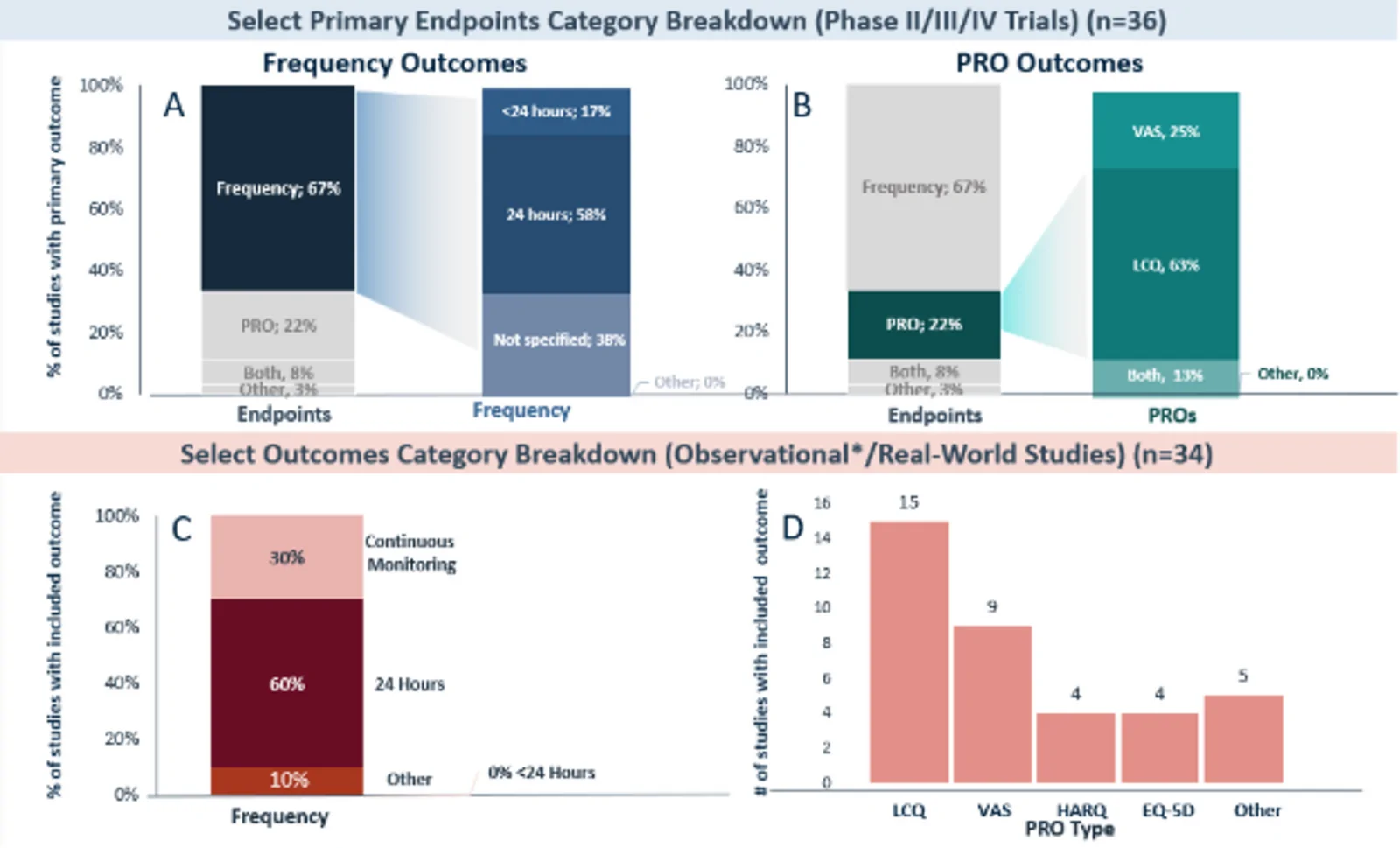
Study endpoints identified in the scoping literature review: **A** summary of objective cough frequency endpoints in clinical trials*; **B** PROs in clinical trials; **C** cough frequency endpoints in observational/real-world studies†; and **D** PRO endpoints in observational/real-world studies. *Observational studies included prospective (*n* = 16), retrospective (*n* = 6) , and other designs (quantitative surveys, registry studies, etc.). ^†^Studies included publications, trials, and abstracts found on PubMed, Clinicaltrials.gov, and grey literature conference searching. EQ-5D, EuroQol 5-Dimension; HARQ, Hull Airway Reflux Questionnaire; LCQ, Leicester Cough Questionnaire; PRO, patient-reported outcome; SGRQ, St George’s Respiratory Questionnaire; VAS, visual analogue scale

Across studies of RCC/UCC, objective cough frequency was consistently included as a primary endpoint (Table 1). Cough frequency was the primary endpoint in 24/36 clinical trials included in the scoping literature review (Fig. 2A). Of these, 24-hour cough frequency was the primary endpoint for 13/24, 3/24 used a duration less than 24 hours, and 8/24 did not specify a time period. For the clinical trials that employed PROs as primary endpoints (8/36), outcomes included the Leicester Cough Questionnaire (LCQ), cough severity visual-analogue scale (CS-VAS), or both (Table 1; Fig. 2B).

**Table 1:** Refractory/unexplained chronic cough study endpoints by publication year

		**Study publication date**		
		**2019–20**	**2021–23**	2024+*
**Interventional**	**Studies (n)**	***N* = 8**	***N* = 11**	***N* = 17**
	*Primary endpoints*			
	Frequency	5	9	10
	Frequency and CS-VAS	2	–	–
	Frequency and LCQ	2	–	1
	No primary efficacy assessment	–	1	–
	LCQ	2	1	2
	CS-VAS	2	–	–
	CSUI	–	1	–
	LCQ and CS-VAS	2	–	1
	*Secondary endpoints*			
	CS-VAS	1	6	10
	LCQ	1	5	8
	HARQ	1	–	–
	Frequency	1	–	3
	CSD	–	3	–
	SF-36	–	1	–
	Incontinence	–	1	–
	Cough intensity	–	–	1
	Presence of wheeze	–	–	1
**Observational**	**Studies (n)**	***N* = 8**	***N* = 20**	***N* = 6**
	*Endpoints*			
	LCQ	3	11	1
	CS-VAS	1	6	2
	HARQ	1	3	–
	Cough symptom score	1	1	–
	Frequency	1	6	3
	EQ-5D	–	4	–
	GAD	–	1	1
	PHQ	–	1	–
	Frequency of nighttime cough†	–	–	1
	Frequency of daytime cough†	–	–	1

^*^Includes protocols/studies in progress; searches may not reveal full extent of endpoints assessed for unpublished studies or studies from conference literature; †qualitative assessment, not through direct measurement

CSD, Cough Severity Diary; CSUI, cough-induced stress urinary incontinence; CS-VAS, cough severity visual analogue scale; EQ-5D, EuroQol 5-Dimension; GAD, Generalized Anxiety Disorder Questionnaire; HARQ, Hull Airway Reflux Questionnaire; LCQ, Leicester Cough Questionnaire; PHQ, Patient Health Questionnaire; SF-36, short form 36-item

Cough frequency as an endpoint was reported in 10 of 34 observational/real-world studies, with 6/10 evaluating 24-h cough frequency and 3/10 using continuous cough monitoring over a period longer than 24 h (Fig. 2C). Frequency of nighttime and daytime cough were only evaluated in one observational/real-world study (Table 1). Observational/real-world studies typically included a greater variety of PROs than clinical trials (Fig. 2D).

Across study types, endpoints included additional measures of QoL (e.g., Generalized Anxiety Disorder Questionnaire, EuroQol 5-Dimension, and the Short Form-36 item questionnaire), as well as specific outcomes that reflect patient burden in RCC/UCC, such as urinary incontinence (e.g., the International Consultation on Incontinence Questionnaire [32]), cough timing (e.g., night vs day), and intensity/severity (Table 1).

#### Devices

Twelve specific devices were identified from prior literature reviews for potential identification in studies of chronic cough and thus RCC/UCC (Table 2). Details of each device vary; however, most were recording devices that record coughs over a specified time followed by manual interpretation by a third-party clinical expert. There was limited device information provided for those studies that captured cough frequency. First-generation digital endpoint devices, such as the LCM and VitaloJAK, were identified in clinical trials, with VitaloJAK being most frequently identified at the time of this study . Also at the time of this study, second-generation devices with additional features have primarily been used in device validation studies. Studies of the Hyfe Cough Monitor, Strados Resp Biosensor and SIVA P3 have highlighted emerging features such as artificial intelligence/machine learning cough identification and continuous monitoring. Additional cough data beyond frequency, including coughing bouts assessments and other measures of cough intensity are also mentioned.

**Table 2:** Devices identified as used to record digital endpoints in studies of cough

Generation	Cough monitor	Hardware	Recording modality	CE mark	510(k)	Identified in studies (trial or observational)
First generation	Cayetano Cough Monitor	Marantz PMD620 handheld recorder	Free-field microphone	No*	No*	No*
	LEOSound lung sound monitor	Custom-built device	3 contact microphones	Yes	No*	No*
	Life Shirt	Custom-built device	Plethysmography, EMG, and electrocardiogram	No*	No*	No*
	LR102	Custom-built device	3 EMG sensors and a contact sound transducer	No*	No*	No*
	PulmoTrack-CC	Custom-built device	2 contact microphones and a pneumogram belt	Yes	No*	No*
	The Hull Automatic Cough Monitor	Sony TCD-D8 Walkman DATi recorder	Free-field microphone	No*	No*	Yes
	The Leicester Cough Monitor	Archos Jukebox Recorder 20	Free-field microphone	Yes	No*	Yes
	VitaloJAK	Purpose-built device	Free-field lapel microphone and contact chest microphone	Yes	Yes†	Yes
Second generation	Hyfe	Smartwatch	Microphone, cough detection algorithm	Yes‡	No^§^	Yes
	SIVA P3	Custom-built device	Sound and vibration sensor, cough identification algorithm	No*	Yes	Yes
	Strados Resp Biosensor	Custom-built device	Multi-Channel MEMS microphone array	Yes	Yes^¶^	Yes
	C-mo	Custom-built device	Surface EMG, field-free microphone, cough identification algorithm	No*	No*	Yes

Non-exhaustive list based on scoping literature review search. *Not identified as of June 2025; ^†^510(k) clearance indicates the device has been demonstrated to be substantially equivalent to a legally marketed device already cleared by the FDA and is not specific to counting coughs; ‡Hyfe has a consumer CE mark; ^§^Cough detection algorithm seeking a de novo classification; ^¶^510(k) clearance indicates the device has been demonstrated to be substantially equivalent to the VitaloJAK and Litmann 3200 devices, already cleared by the FDA

EMG, electromyography; FDA, US Food and Drug Administration; MEMS, microelectromechanical system

## Discussion

In this scoping literature review (2019–2024), we found that objective, 24-hour cough frequency remains the most commonly used endpoint in studies of chronic cough. Recently, this has been combined with a broader range of both objective and PRO measures to better capture the overall burden of cough. In observational/real-world studies, PROs were more commonly used as outcomes compared with objective 24-hour cough frequency. Further digital endpoints and PROs are being developed as research into RCC/UCC continues to expand.

Cough severity is made up of many components, as evidenced by the only moderate correlation between objective cough frequency and currently available PROs [24, 26], underscoring that cough severity is made up of many components. A previous systematic literature review identified more than 40 different components of ‘cough severity’ that could influence outcomes [33]. These insights highlight the importance of evaluating RCC/UCC holistically using new and existing PROs, 24-hour cough frequency, and additional components of cough to more fully capture its effect on patients, including overall QoL. For novel measures such as cough bouts, there is no current consensus on the definition of how coughs should be clustered into cough bouts [3]. Digital measures of cough and emerging PRO endpoints might support better capturing of the impact of RCC/UCC on patients.

A specific device was reported in 12 of the 24 identified clinical trials that measured objective cough frequency. VitaloJAK, a first-generation device,was the most frequently identified in clinical trial settings, while second-generation devices were mostly reported in ongoing device validation studies. When considering devices to measure cough, sponsors must meet regulatory, technical, and scientific requirements that ensure the data are valid, reliable, and clinically meaningful [29]. Thus far, cough monitoring devices that meet criteria for data collection in clinical trials have automatically recorded coughs over a defined period of time and are the preferred approach for reliable measurement but remain subject to human validation [34]. The use of AI without human involvement is an emerging trend, with the potential benefit of automating the steps involved in accurate cough counting [35, 36]. In-depth research is required to establish the accuracy and reliability of these processes for new approaches to digital endpoints [29].

The future value of digital biomarkers and endpoints in RCC/UCC extends beyond replacing manual cough counts with automated measurement. Objective digital measures and PROs should be considered as complementary tools for assessing disease burden [27, 37]. While digital measures characterize what occurred (e.g., cough frequency and bout structure, cough-free time, nocturnal burden, trigger responsiveness, and functional disruption), PROs remain essential for understanding how cough affects patients (e.g., sleep, communication, social participation, emotional well-being, and patient QoL). Increased measurement density should not be equated with increased evidentiary value; the usefulness of a digital measure will depend on its reliability, interpretability, tolerability to participants, resistance to missingness and algorithmic drift, and alignment with an aspect of health that matters to patients [27, 29, 38].

As technologies emerge and become more accessible, they may also transform routine clinical care. Rather than relying on retrospective patient recall between appointments, clinicians could review cough trends, flare-ups, medication adherence, trigger exposure, and treatment response before consultations, allowing visits to focus more on disease management rather than history taking [29, 37, 38]. For patients, continuous objective monitoring may validate their lived experience of chronic cough, reduce uncertainty about their progress, improve understanding of cough triggers and treatment responses, and facilitate more informed discussions about QoL. Continuous remote monitoring may also reduce the need for repeated in-person assessments while still providing rich longitudinal information for clinicians [29, 37].

In drug development, digital endpoints and more sensitive PROs may provide more ecologically valid and precise estimates of treatment effect, facilitate decentralized participation, and support multidimensional responder definitions [29, 38]. Additionally, they may improve sensitivity for detecting clinically meaningful changes that may not be reflected by average cough frequency alone [38]. Future development programs may prospectively evaluate the relationship between digital cough measures and patient-perceived benefit, including whether improvements exceed meaningful within-patient change thresholds and are sustained across relevant daily contexts.

Phase 4 and other longitudinal real-world studies may further expand the role of digital measurement by evaluating durability, adherence, comparative effectiveness, healthcare utilization, productivity, caregiver burden, and treatment-effect heterogeneity [38–40]. Such longitudinal data may also improve the understanding of the natural history of RCC/UCC, enable digital phenotyping, identify potential predictors of treatment response and support more personalized treatment strategies [29, 38].

Realizing these opportunities will require rigorous approaches to confounding, selection bias, data provenance, privacy, interoperability, equitable access, and algorithm governance [29, 38]. Continued collaboration between researchers, clinicians, patients, regulators, and industry will be important to establish consensus definitions, transparent validation standards, patient-informed measure development, algorithm version control, and early regulatory engagement [27, 41]. Rather than pursuing multiple technically derivable features, future research should prioritize a parsimonious set of reproducible measures that meaningfully inform clinical and drug-development decision-making [38].

However, there remain potential barriers to the adoption of digital endpoints for cough in clinical trials and real-world studies. For example, specific requirements may be needed for digital endpoints suitable for different conditions (e.g., RCC/UCC, idiopathic pulmonary fibrosis) [37]. There is also a need to build support and understanding of these measures. Although objective measures have value in research settings, their role in routine clinical practice is still evolving. Objective and subjective measures of cough should be viewed as complementary tools to support the overall clinical assessment. Greater clarity and direction from treatment guidelines and recommendations could support establishment of best practices for measurement of digital endpoints in RCC/UCC.

A limitation of this study is that scoping literature reviews are less comprehensive than systematic literature reviews. Scoping reviews do not incorporate formal assessment of the risk of bias, and have a reduced capacity to generate definitive answers to a specific research question. While the search terms included ‘chronic cough’ and studies were included based on participants meeting the clinical definitions of RCC or UCC, excluding search terms such as ‘idiopathic chronic cough’ or ‘cough hypersensitivity’ may have limited the identification of studies.

## Conclusions

Across clinical trials of RCC/UCC, objective measures of cough frequency remain one of the most common cough endpoints, while observational/real-world studies make use of a wider variety of endpoints. In many ways, RCC/UCC is particularly well suited to digital medicine because the disease is defined by a symptom that is frequent, objectively measurable, and has profound impacts on daily functioning. In the future, as wearable sensors, AI-based cough detection, and validated digital biomarkers and PROs continue to mature, they have the potential to reshape not only how new therapies are evaluated, but also how clinicians diagnose, monitor, and personalize care for patients living with chronic cough diseases such as RCC/UCC. There is an ongoing need to connect objective measures to meaningful changes for patients, with studies including endpoints that consistently capture improvements in QoL. New approaches to the identification of additional digital endpoints and PROs are being developed as research in cough continues to expand.

## Supplementary Information

Below is the link to the electronic supplementary material.

Supplementary file1.

## Data Availability

Data sharing is not applicable to this article as no datasets were generated or analyzed during the current study.
