# Supplementary material for: A Scoping Literature Review of Digital Measures and Patient-Reported Outcomes for Refractory/Unexplained Chronic Cough: Assessment of Clinical Trials and Real-World Clinical Practice
Source: Lung. 2026 Sep 10;204(1):66. doi: 10.1007/s00408-026-00928-5 (PMC13562093; doi:10.1007/s00408-026-00928-5)
Supplement: Supplementary file 1 [file 408_2026_928_MOESM1_ESM.docx]

**Supplement**

## Supplementary results

**Supplementary Table 1:** Literature search results for clinical trials*

| NCT Number | Study Title | Study Type (Study design, phase) | Study Start | Study End | Corresponding Publication |
| --- | --- | --- | --- | --- | --- |
| NCT02233699 | A Study to Assess the Efficacy of XEN-D0501 in Reducing the Cough Frequency in Patients With Chronic Idiopathic Cough | Phase II | 2015 | 2018 | Belvisi, et al. Am J Crit Care Med 2017 |
| NCT02993822 | A Dose-ranging Study of Orvepitant in Patients With Chronic Refractory Cough | Phase II | 2017 | 2019 | – |
| NCT03622216 | A Dose Escalation Study of Bradanicline in Refractory Chronic Cough | Phase II | 2018 | 2019 | Kanemitsu et al. Euro Respir J. 2020 |
| NCT03372603 | A Study to Assess the Effectiveness and Side Effects of GSK2798745 in Participants With Chronic Cough | Phase II | 2018 | 2018 | – |
| NCT03282591 | Study of the Efficacy, Safety, and Tolerability of Serlopitant for the Treatment of Refractory Chronic Cough | Phase II | 2018 | 2018 | – |
| NCT02479074 | The Utility of FeNO in the Differential Diagnosis of Chronic Cough | Phase IV | 2018 | 2017 | Sadeghi, et al. Lung. 2018 |
| NCT04525885 | A Study of Gefapixant (MK-7264) in Adult Participants With Chronic Cough (MK-7264-030)-China Extension | Phase III | 2019 | 2022 | – |
| NCT03235466 | A Prospective Trial of Behavioral Therapy for Chronic Cough | Interventional - no phase | 2019 | 2021 | – |
| NCT01252225 | Lidocaine: Effect of Lidocaine in Chronic Cough | Phase IV | 2019 | 2020 | Abdulqawi, et al. J Allerg Clin Immunol. 2021 |
| NCT03979638 | A Dose Escalation Study of BLU-5937 in Unexplained or Refractory Chronic Cough | Phase II | 2019 | 2020 | Chauret et al. Am J Respir Crit Care Med 2023 |
| NCT04193176 | Efficacy and Safety of Gefapixant in Women with Chronic Cough and Cough-Induced Stress Urinary Incontinence: a Phase 3b, Randomised, Multicentre, Double-Blind, Placebo-Controlled Trial | Phase III | 2020 | 2022 | Birring, et al. Lancet Respir Med 2024 |
| NCT04193202 | Efficacy and Safety of Gefapixant (MK-7264) in Adult Participants With Recent Onset Chronic Cough (MK-7264-043) | Phase III | 2020 | 2021 | McGarvey, et al. Lung 2024 |
| NCT04678206 | Evaluation of the Efficacy and Safety of BLU-5937 in Adults With Refractory Chronic Cough | Phase II | 2020 | 2021 | Sher, et al. J Allergy Clin Immunol 2023 |
| NCT04562155 | Clinical Study to Evaluate the Efficacy and Safety of Three Different Doses of BAY1817080 Compared to Placebo in Patients With Chronic Cough (PAGANINI) | Phase II | 2020 | 2021 | Dicpinigaitis, et al. Lung, 2023 |
| NCT04110054 | Evaluation of S-600918 in Adults With Refractory Chronic Cough | Phase II | 2020 | 2020 | McGarvey, et al. Lung, 2023 |
| NCT03449147 | A Study of Gefapixant (MK-7264) in Adult Participants With Chronic Cough (MK-7264-030) | Phase III | 2020 | 2020 | McGarvey, et al. Lancet 2022 |
| NCT03449134 | A Study of Gefapixant (MK-7264) in Adult Participants With Chronic Cough (MK-7264-027) | Phase III | 2020 | 2020 | McGarvey et al. Lancet 2022 |
| NCT05273190 | Stimulation of the Larynx to Treat Unexplained Chronic Cough | Interventional - no phase | 2021 | 2027 | Misono, et al. The Laryngoscope 2024 |
| NCT04866563 | A Study of Efficacy and Safety of AX-8 in Chronic Cough | Phase II | 2021 | 2025 | – |
| NCT05110144 | Efficacy of Two Doses of Duloxetine and Amitriptyline in Subjects With Refractory Chronic Cough | Phase II | 2021 | 2025 | Agarwal, et al. Chest 2023 |
| NCT04765722 | Mepolizumab for the Treatment of Chronic Cough With Eosinophilic Airways Diseases | Phase IV | 2021 | 2024 | – |
| NCT05226299 | Cough Desensitization Therapy: Pilot 2 (CDTPilot2) | Phase I | 2021 | 2022 | – |
| NCT04861155 | Validation and Evaluation of a Novel Cough Detection Device | Interventional - no phase | 2021 | 2021 | Kuhn, et al. ERJ Open Res 2023 |
| NCT02482818 | Efficacy of Pregabalin on Chronic Cough | Phase II | 2021 | 2021 | – |
| NCT05600777 | A 24-Week Study of the Efficacy and Safety of BLU-5937 in Adults With Refractory Chronic Cough (CALM-2) | Phase III | 2022 | 2026 | – |
| NCT05599191 | A 52-Week Study of the Efficacy and Safety of BLU-5937 in Adults with Refractory Chronic Cough | Phase III | 2022 | 2025 | – |
| NCT05392192 | A Clinical Trial to Evaluate the Safety and Efficacy in Subjects With Chronic Cough | Phase II | 2022 | 2023 | – |
| N/A | A double-blind randomised placebo-controlled trial investigating the effects of lesogaberan on the objective cough frequency and capsaicin-evoked coughs in patients with refractory chronic cough | Interventional - no phase | 2022 | 2023 | Badri, et al. ERG Open Res 2022 |
| NCT05989698 | C-mo System 1.0's Validation - Cough Monitoring | Interventional - no phase | 2023 | 2025 | – |
| NCT05713019 | ATP and P2X3 Receptor in Chronic Cough | Interventional - no phase | 2023 | 2024 | – |
| NCT05689307 | Validation Study With a Non-CE Marked Medical Device (MD) | Interventional - no phase | 2023 | 2024 | – |
| NCT05660850 | A Study To Evaluate The Efficacy, Safety, Pharmacokinetics, And Pharmacodynamic Effects Of GDC-6599 In Patients With Chronic Cough | Phase II | 2023 | 2024 | – |
| NCT06706765 | Group Telehealth Behavioral Cough Suppression Therapy | Phase I | 2024 | 2025 | – |
| NCT06246565 | A Study of HS-10383 in Chinese Adult Subjects With Refractory or Unexplained Chronic Cough (RUCC) | Phase II | 2024 | 2025 | – |
| NCT06504446 | Study to Assess the Efficacy, Safety, and Tolerability of NOC-110 in Adults With Refractory or Unexplained Chronic Cough | Phase II | 2024 | 2025 | – |
| NCT06378671 | Single-center Clinical Study on the Safety and Effect of Bronchoscopic Cryotherapy in Patients With Chronic Cough | Interventional - no phase | 2024 | 2024 | – |

*The corresponding publications are representative of publications for which data were reviewed. Some studies resulted in multiple publications; in these cases, additional data not included in this review.

**Supplementary Table 2.** Literature search results for non-RCTs

| Title | Citation |
| --- | --- |
| An Observational Study Using Novel Questionnaire to Characterize Cough Phenotypes in Patients With Chronic Cough (NCT06376448) | 2025 [trial ongoing; no publication available] |
| Prospective Study on the Evaluation, Patient Reported Outcomes and Effectiveness of Treatment for Chronic Cough (NCT04758351) | 2025 [trial ongoing; no publication available] |
| Longitudinal cough frequency monitoring in persistent coughers: daily variability and predictability | Chung, et al. Lung 2024 |
| Patient preferences for the treatment of chronic cough: a discrete choice experiment | Swaminathan, et al. BMJ Open Resp Res 2024 |
| Development of the mcmaster cough severity questionnaire (MCSQ): a cough severity instrument for patients with chronic cough | Kum, et al. ATS, 2024 |
| New insights into refractory chronic cough and unexplained chronic cough: a 6-year ambispective cohort study | Zhang, et al. Allergy Asthma Immunol Res 2023 |
| Baseline cohort profile of the korean chronic cough registry: a multicenter, prospective, observational study | Jo, et al. Lung 2023 |
| Predictors of treatment response to pregabalin in unexplained or refractory chronic cough | Saint-Pierre, et al. Respir Med 2023 |
| Gabapentin for chronic refractory cough: a system review and meta-analysis | Xie, et al. Heliyon 2023 |
| Anxiety and depression diagnoses and the cough severity index: a retrospective study | Hari, et al. Ear Nose Throat J 2023 |
| Interaction effect of chronic cough and ageing on increased risk of exacerbation in patients with asthma: a prospective cohort study in a real-world setting | Jin, et al. ERJ Open Res 2023 |
| Feasibility and utility of a smartphone application-based longitudinal cough monitoring in chronic cough patients in a real-world setting | Lee, et al. Lung 2023 |
| Demographic, clinical, and patient-reported outcome data from 2 global, phase 3 trials of chronic cough | Dicpinigaitis, et al. Ann Allergy Asthma Immunol 2023 |
| Chronic cough-related differences in brain morphometry in adults: a population-based study | Arinze, et al. Chest 2023 |
| Health-related quality of life and its determinants in chronic cough: the Korean chronic cough registry study | Kang, et al. Allergy Asthma Immunol Res 2023 |
| Antitussive efficacy of the current treatment protocol for refractory chronic cough: our real-world experience in a retrospective cohort study | Zhang, et al. Ther Adv Respir Dis 2023 |
| A real world study of cough burden and quality of life of UK patients who have undergone evaluation for chronic cough | McGarvey, et al. Curr Med Res Opin 2023 |
| Effects of pregabalin as a neural pathway inhibitor for the treatment of resistant subacute and chronic cough: a pilot clinical trials study | Adeli, et al. Rev Recent Clin Trials 2023 |
| Subcutaneous octreotide as a therapeutic option for refractory chronic cough: subjective response pattern and adverse effects from a retrospective single-centre case series | Brindle, et al. ERJ Open Res 2023 |
| Quality of life in adults with chronic cough: a mixed methods study informing the development of a quantitative patient preference study | Coles, et al. The Patient 2023 |
| Healthcare utilization and costs in chronic cough | Cho, et al. Curr Med Res Opin 2022 |
| Impact of productive and dry chronic cough on mortality in the Canadian longitudinal study on aging (CLSA) | Satia, et al. J Thorac Dis 2022 |
| Clinical characteristics of patients with chronic cough in Guangdong, China: a multicenter descriptive study | Lai, et al. BMC Pulm Med 2021 |
| Feasibility and clinical utility of ambulatory cough monitoring in an outpatient clinical setting: a real-world retrospective evaluation | Vertigan, et al. ERJ Open Res 2021 |
| Long-term treatment outcomes after behavioral speech therapy for chronic refractory cough | Wright, et al. Lung 2021 |
| Cough persistence in adults with chronic cough: a 4-year retrospective cohort study | Kang, et al. Allergol Int 2020 |
| Prevalence and characteristics of chronic cough in adults identified by administrative data | Zeiger, et al. Perm J 2020 |
| Quantitative measurement properties and score interpretation of the cough severity diary in patients with chronic cough | Nguyen, et al. Ther Adv Respir Dis 2020 |
| Idiopathic chronic productive cough and response to open-label macrolide therapy: an observational study | Martin, et al. Respirology 2019 |
| Are chronic cough and laryngopharyngeal reflux more common in obstructive sleep apnea patients? | Gouveia, et al. Laryngoscope 2019 |
| Efficacy and predictors of response to inhaled corticosteroid treatment for chronic cough | Hong, et al. Korean J Intern Med 2019 |
| Randomised clinical trial: gabapentin vs baclofen in the treatment of suspected refractory gastro-oesophageal reflux-induced chronic cough | Dong, et al. Aliment Pharmacol Ther 2019 |
| A Wireless Bed Sensor for Monitoring Coughs (NCT03177694) | 2019 [no publication available] |
| Prevalence of Anxiety as a Variable in Treatment Outcomes for Individuals With Chronic Refractory Cough | Wright, et al. Am J Speech Lang Pathol 2024 |

**Supplementary methods: PRISMA-ScR checklist**

**Preferred Reporting Items for Systematic reviews and Meta-Analyses extension for Scoping Reviews (PRISMA-ScR) Checklist**

| **SECTION** | **ITEM** | **PRISMA-ScR CHECKLIST ITEM** | **REPORTED ON PAGE #** |
| --- | --- | --- | --- |
| **TITLE** | | | |
| Title | 1 | Identify the report as a scoping review. | Title |
| **ABSTRACT** | | | |
| Structured summary | 2 | Provide a structured summary that includes (as applicable): background, objectives, eligibility criteria, sources of evidence, charting methods, results, and conclusions that relate to the review questions and objectives. | Abstract |
| **INTRODUCTION** | | | |
| Rationale | 3 | Describe the rationale for the review in the context of what is already known. Explain why the review questions/objectives lend themselves to a scoping review approach. | Intro/Methods |
| Objectives | 4 | Provide an explicit statement of the questions and objectives being addressed with reference to their key elements (e.g., population or participants, concepts, and context) or other relevant key elements used to conceptualise the review questions and/or objectives. | Intro/Methods |
| **METHODS** | | | |
| Protocol and registration | 5 | Indicate whether a review protocol exists; state if and where it can be accessed (e.g., a Web address); and if available, provide registration information, including the registration number. | Methods |
| Eligibility criteria | 6 | Specify characteristics of the sources of evidence used as eligibility criteria (e.g., years considered, language, and publication status), and provide a rationale. | Methods/Supplement |
| Information sources* | 7 | Describe all information sources in the search (e.g., databases with dates of coverage and contact with authors to identify additional sources), as well as the date the most recent search was executed. | Methods/Supplement |
| Search | 8 | Present the full electronic search strategy for at least 1 database, including any limits used, such that it could be repeated. | Supplement |
| Selection of sources of evidence^†^ | 9 | State the process for selecting sources of evidence (i.e., screening and eligibility) included in the scoping review. | Methods/Supplement |
| Data charting process^‡^ | 10 | Describe the methods of charting data from the included sources of evidence (e.g., calibrated forms or forms that have been tested by the team before their use, and whether data charting was done independently or in duplicate) and any processes for obtaining and confirming data from investigators. | Supplement |
| Data items | 11 | List and define all variables for which data were sought and any assumptions and simplifications made. | Methods |
| Critical appraisal of individual sources of evidence^§^ | 12 | If done, provide a rationale for conducting a critical appraisal of included sources of evidence; describe the methods used and how this information was used in any data synthesis (if appropriate). | N/A |
| Synthesis of results | 13 | Describe the methods of handling and summarizing the data that were charted. | Methods |
| **RESULTS** | | | |
| Selection of sources of evidence | 14 | Give numbers of sources of evidence screened, assessed for eligibility, and included in the review, with reasons for exclusions at each stage, ideally using a flow diagram. | Results/Fig. 1 |
| Characteristics of sources of evidence | 15 | For each source of evidence, present characteristics for which data were charted and provide the citations. | Supplement |
| Critical appraisal within sources of evidence | 16 | If done, present data on critical appraisal of included sources of evidence (see item 12). | N/A |
| Results of individual sources of evidence | 17 | For each included source of evidence, present the relevant data that were charted that relate to the review questions and objectives. | Results |
| Synthesis of results | 18 | Summarize and/or present the charting results as they relate to the review questions and objectives. | Results/Table 1/Fig. 2 |
| **DISCUSSION** | | | |
| Summary of evidence | 19 | Summarize the main results (including an overview of concepts, themes, and types of evidence available), link to the review questions and objectives, and consider the relevance to key groups. | Results/ Discussion |
| Limitations | 20 | Discuss the limitations of the scoping review process. | Discussion |
| Conclusions | 21 | Provide a general interpretation of the results with respect to the review questions and objectives, as well as potential implications and/or next steps. | Discussion |
| **FUNDING** | | | |
| Funding | 22 | Describe sources of funding for the included sources of evidence, as well as sources of funding for the scoping review. Describe the role of the funders of the scoping review. | Statements and declarations |

JBI, Joanna Briggs Institute; PRISMA-ScR, Preferred Reporting Items for Systematic reviews and Meta‑Analyses extension for Scoping Reviews.

*Where sources of evidence (see second footnote) are compiled from, such as bibliographic databases, social media platforms, and Web sites.

^†^A more inclusive/heterogeneous term used to account for the different types of evidence or data sources (e.g., quantitative and/or qualitative research, expert opinion, and policy documents) that may be eligible in a scoping review as opposed to only studies. This is not to be confused with information sources (see first footnote).

^‡^The frameworks by Arksey and O’Malley (6) and Levac and colleagues (7) and the JBI guidance (4, 5) refer to the process of data extraction in a scoping review as data charting.

^§^The process of systematically examining research evidence to assess its validity, results, and relevance before using it to inform a decision. This term is used for items 12 and 19 instead of "risk of bias" (which is more applicable to systematic reviews of interventions) to include and acknowledge the various sources of evidence that may be used in a scoping review (e.g., quantitative and/or qualitative research, expert opinion, and policy document).
